# Metabolic requirements of Th17 cells and of B cells: Regulation and defects in health and in inflammatory diseases

**DOI:** 10.3389/fimmu.2022.990794

**Published:** 2022-10-14

**Authors:** Jonas Bystrom, Taher E. Taher, Sian M. Henson, David J. Gould, Rizgar A. Mageed

**Affiliations:** ^1^ Centre for Translational Medicine and Therapeutics, William Harvey Research Institute, Queen Mary University of London, London, United Kingdom; ^2^ Institute of Immunology and Immunotherapy, College of Medical and Dental Sciences, University of Birmingham, Birmingham, United Kingdom; ^3^ Centre for Biochemical Pharmacology, William Harvey Research Institute, Queen Mary University of London, London, United Kingdom

**Keywords:** metabolism, Th17 cells, B cells, mTORC, OXPHOS, epigenetics, autoimmunity

## Abstract

The immune system protects from infections and cancer through complex cellular networks. For this purpose, immune cells require well-developed mechanisms of energy generation. However, the immune system itself can also cause diseases when defective regulation results in the emergence of autoreactive lymphocytes. Recent studies provide insights into how differential patterns of immune cell responses are associated with selective metabolic pathways. This review will examine the changing metabolic requirements of Th17 cells and of B cells at different stages of their development and activation. Both cells provide protection but can also mediate diseases through the production of autoantibodies and the production of proinflammatory mediators. In health, B cells produce antibodies and cytokines and present antigens to T cells to mount specific immunity. Th17 cells, on the other hand, provide protection against extra cellular pathogens at mucosal surfaces but can also drive chronic inflammation. The latter cells can also promote the differentiation of B cells to plasma cells to produce more autoantibodies. Metabolism-regulated checkpoints at different stages of their development ensure the that self-reactive B cells clones and needless production of interleukin (IL-)17 are limited. The metabolic regulation of the two cell types has some similarities, e.g. the utility of hypoxia induced factor (HIF)1α during low oxygen tension, to prevent autoimmunity and regulate inflammation. There are also clear differences, as Th17 cells only are vulnerable to the lack of certain amino acids. B cells, unlike Th17 cells, are also dependent of mechanistic target of rapamycin 2 (mTORC2) to function. Significant knowledge has recently been gained, particularly on Th17 cells, on how metabolism regulates these cells through influencing their epigenome. Metabolic dysregulation of Th17 cells and B cells can lead to chronic inflammation. Disease associated alterations in the genome can, in addition, cause dysregulation to metabolism and, thereby, result in epigenetic alterations in these cells. Recent studies highlight how pathology can result from the cooperation between the two cell types but only few have so far addressed the key metabolic alterations in such settings. Knowledge of the impact of metabolic dysfunction on chronic inflammation and pathology can reveal novel therapeutic targets to treat such diseases.

## Introduction

T and B lymphocytes play central and complementary roles in protecting from infections and cancer. Gene rearrangements in these cells generate an extraordinarily diverse array of antigen-specific receptors, the T- and B-cell receptors (TCR and BCR). The two cell lineages originate in the bone marrow, where B cells generate their BCR. T-cell progenitors migrate to the thymus to undergo TCR gene rearrangements and a programmed range of selective processes. Naive T and B cells then migrate from these primary lymphoid organs and circulate through the blood and the lymphatic system to encounter their target antigens, become activated, proliferate, and differentiate to effector cells. Pathways of T- and B-cell activation depends on their target antigens, the microenvironment, and how to most efficiently conferring effective immunity.

In the process of mounting immunity, naive B cells are selected, activated, and differentiate to antibody-producing plasma cells or memory cells dependent on the type of antigen, availability of T-cell help, and also of cytokines produced. Naive helper T cells (Th cells) can differentiate to distinct functional subsets dependent on the type of antigen, antigen-presenting cell type, and cytokines produced. These Th subsets include Th17, Th1, Th2, induced regulatory T (iTreg), and follicular helper T (Tfh) cells. Differentiation of T cells to distinct functional subsets is associated with the upregulation of unique transcription factors. These are T-box expressed in T cells (T-bet) in Th1 cells, GATA-binding protein 3 (GATA-3) in Th2 T cells, and RAR-related orphan receptor γt (RORγt) in Th17 cells. Naive T cells are induced to differentiate to Th17 cells in the presence of interleukin (IL)-1β, IL-6, IL-23, and transforming growth factor-β (TGFβ) leading to upregulation of transcription factors RORγt, basic leucine zipper ATF-like (BATF) and signal transducer and activator of transcription 3 (STAT3) (1). Th1 cells protect from intracellular microorganisms and viruses, while Th2 protects from parasites and helminths. Th17 cells, combat extracellular bacterial and fungal infections primary on mucosal membranes. In addition to these main T-cell subsets, other T-cell subsets exist, and these contribute to the regulation and refinement of immunity. Natural regulatory T cells (nTregs) are generated and educated in the thymus to regulate immunity and limit autoimmune reactions (2, 3). In addition, chronic antigen stimulation in the periphery, in the presence of TGFβ leads to forkhead box P3 (FOXP3) upregulation in T cells to promote the differentiation of T cells to induced Tregs (iTregs) (3). Tfh cells promote B-cell activation and differentiation in germinal centers (GCs). Tfh cells are characterized by the expression of the transcription factor Bcl-6, and differentiation is facilitated by IL-21. Follicular regulatory T (Tfr) cells, in contrast, prevent Tfh-cell activity and suppress autoreactivity (3).

## Metabolic requirements of T and B cells

Over the last decade, it has emerged that throughout their life spans, lymphocytes differentiate and function using distinct metabolic pathways, directed by specific functional needs at each stage of their development, the microenvironment, and the availability of nutrients and oxygen (O_2_) tension (4). Basic metabolic pathways in these cells involve glycolysis and the pentose-phosphate pathway (PPP) that are key for their effector functions. During glycolysis, glucose is actively transported to the cytoplasm and metabolized by a set of 10 enzymes to generate energy-rich pyruvate and NAD^+^. In proliferating T cells that rely on glycolysis for their energy needs, NAD^+^ is reduced to NADH with lactate as a by-product. Low O_2_ tension in certain niches, such as the bone marrow, the light zone (LZ) of GCs of B-cell follicles, and the mucosa, activates the transcription factor hypoxia-inducible factor 1α (HIF1α) that regulates genes that control glycolysis (5). Mechanistic target of rapamycin (mTOR) is a large protein complex located at the endosome of lymphocytes. The subcomponent Raptor associates with mTOR to form mTORC1, while Rictor associates with mTOR to form mTORC2. mTORC1 senses amino acid availability, regulates cell differentiation to effector cells, and determines the selection, or death of B cells in the LZ of GCs (6–8). mTORC2 is regulated *via* phosphatidylinositol 3-kinase (PI3K) and growth factor signaling and promotes the differentiation of Th cells to Th2 cells. mTORC2 also cooperates with mTORC1 in B-cell activation (9). AMP-activated protein kinase (AMPK), which is localized to the cytoplasm or the lysosomes, is a crucial energy sensor that reduces cellular activity, augments fatty acid oxidation (FAO), and maintains quiescence of cells (7, 10, 11).

The glycolysis in the cytoplasm generates a limited amount of ATP and substrates for amino acid, nucleotide, and fatty acid biosynthesis as well as pyruvate for the more efficient energy producers, the mitochondria. In the mitochondrial compartment, the tricarboxylic acid (TCA) cycle is replenished by β-fatty acid oxidation, pyruvate and imported amino acids in what is called anaplerotic reactions. Pyruvate, which is transported from the cytoplasm, is converted in the mitochondria to TCA substrate acetyl-coenzyme A (acetyl-CoA) by the pyruvate dehydrogenase (PDH1). The TCA cycle provides substrates for the mitochondrial inner membrane-residing electron transport chain (ETC). The resulting conversion of O_2_ to H_2_O and NADH to NAD^+^ generates ATP in what is known as oxidative phosphorylation (OXPHOS). During this process, the ETC transports protons to the intermembrane space, thereby establishing mitochondrial membrane potential (ΔΨm). This potential is essential for the function and metabolism of effector cells and for their early progenitors to develop in the bone marrow (12). During activation, the availability/lack of nutrients and O_2_ tension influence ΔΨm. The mitochondria also have a role in Ca^2+^ homeostasis, with the ion being transported from the endoplasmic reticulum (ER) and from outside of the cell and, therefore, influencing ΔΨm in the process. TCR and BCR signaling results in increased mitochondrial Ca^2+^ (13). Metabolites generated through anaplerosis contribute to the biomass but also influence epigenetic regulators and gene transcription (see **Table 1** for Th17 cells). The TCA cycle and OXPHOS provide basal functional requirements to all cells and are the only pathways that drive naive and non-activated memory T, B, and Tregs. It is now widely recognized that changes to the metabolic properties of different cell subsets are of fundamental importance to the regulation of the cell-specific transcriptional programs and effector functions.

**Table 1:** Metabolic alterations resulting in epigenetic changes to Th17 cells.

Metabolic alteration	Effect	Epigenetic / transcription factor change	Th17/Treg	Reference
Glutamine intake	↑α-ketoglutarate and 2-HG	↑Methylation of the *Foxp3* promoter	Treg ↓	(14)
Lack of glutamine	↑ ROS	↑ H3K27 trimethylation globally	Th17 ↓	(15)
Lack of methionine	↓ SAM	↓ H3K4 methylation, promoter of genes involved in Th17 cell differentiation; *Il17 and Batf* and cell cycle	Th17 ↓	(16)
High (GLS1 and) Acetyl-CoA		↑ Acetylation of H3K9Ac and H3K27Ac in the *Il17* promoter	Th17 ↑	(17)
Lack of MTHFD2	↓ Succinate, fumarate	↓ DNA demethylation in the *Foxp3* promoter	Treg ↑	(18)
Inhibition of polyamine		FOXP3 ↑	Th17 ↓/ Treg ↑	(19)
metabolism				
HIF1α		1) Associate with RORγt and p300 on the *Il17* promoter 2) Binds FOXP3	Th17↑ / Treg ↓	(20)
Pentanoate from SFB		Histone deacetylase inhibitor	Th17 ↓	(21)

GLS, glutaminase 1; GOT1, glutamate oxaloacetate transaminase 1; 2-HG, 2-hydroxyglutarate; MTHFD2, bifunctional methylenetetrahydrofolate dehydrogenase/cyclohydrolase; SAM, S-adenosylmethionine; SFB, segmented filamentous bacteria. ↑: increased, ↓: decreased.

The focus of this review is on the changing metabolic requirements of Th17 cells and of B cells at different stages of their development and activation. These two cell types have been selected because of their role in promoting chronic inflammation that underpins pathology in most chronic diseases. Generally, metabolism-regulated checkpoints at different stages of lymphocyte development are instilled to prevent the emergence of self-reactive B- and T-cell clones (**Table 2** for B cells). Metabolic regulation of B cells and Th17 cells have similarities. For example, both cell types utilize HIF1α during low oxygen tension. In addition, both cells express the anti-inflammatory adenosine-sensing CD73 molecule (4, 5, 29). The two cell types, nevertheless, have differences too. As will be discussed later on, Th17 cells are particularly vulnerable to the lack of certain amino acids. This latter issue has not been reported in B cells (15, 30). B cells, unlike Th17 cells, are dependent on mTORC2 for their functions (9). Although lymphocyte activation following TCR and BCR engagements induce metabolic changes required for immunity against pathogens, it is increasingly recognized that metabolic dysregulation of Th17 cells or B cells relates to the development of autoimmune diseases due to defective tolerance (17, 31). Disease-associated genetic variations might be one cause of metabolism and epigenetic dysregulation in these cells (32, 33). Recent reports have highlighted autoimmune pathology-inducing interactions between the two cell types, but only a few have so far assessed altered metabolic properties of the two cell types during such scenarios (34–36).

**Table 2:** Metabolism at B-cell stages where clones with low antigen specificity or self-reactivity are excluded.

	Exclusion selection due to self reactivity / low antigen specificity	Metabolism type	Reference
**Bone marrow**			
Large pre-B cells	Autoreactivity of the IgH chain	Glycolysis ↑, OXPHOS ↑ Low O_2_, HIF1α ↑	(22, 23)
Immature B cells	Autoreactivity of BCR	Glycolysis ↓, OXPHOS ↓ FOXO1↑	(23, 24)
**Spleen**			
Transitional B cell	Autoreactivity of BCR	Glycolysis ↑, OXPHOS ↑	(7)
**Spleen and lymph nodes**			
Activated B cell	BCR not recognized via CD40 or TLR	Glycolysis ↑, OXPHOS ↑	(25, 26)
Light Zone B cell	Competition for BCR selection, IgH class switch	GSK3 ↑, Low O_2_, HIF1α ↑	(5, 8, 27, 28)

↑: increased, ↓: decreased.

## Metabolism in T cells

T-cell progenitors are produced in the bone marrow. They migrate to the thymus where they undergo development, including rearrangement of their TCR genes, positive recognition of the major histocompatibility complex II, and tolerance to prevent self-reactivity. During these developmental phases, the functioning metabolic pathway changes with high glycolysis and OXPHOS during TCR arrangement followed by low metabolic activity during positive and negative selections (37). Thymic outputs are highest during childhood, continue at a slower rate in adolescence, and are then much reduced after the third decade of life, and these changes are reflected by changes in thymocyte metabolism (38).

**Figure 1:**
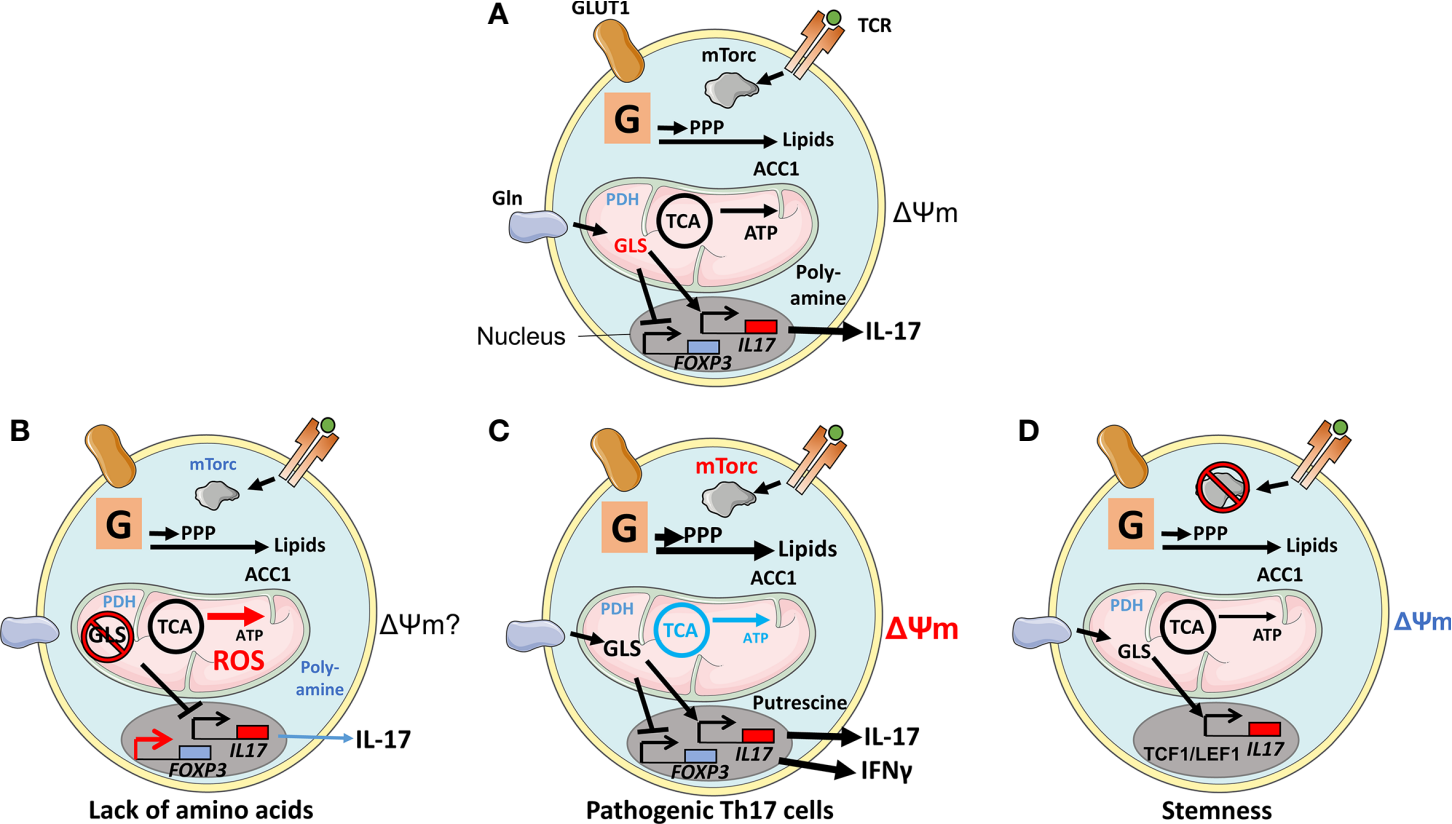
Physiological and experimentally induced metabolic states of Th17 cells. **(A)** After T cell receptor (TCR) engagement, Glucose transporter 1(GLUT1) is upregulated in support of glycolysis (denoted as a G within a square). TCR engagement also augments mechanistic target of rapamycin (mTORC1) activity *via* Akt/PI3K, leading to cell differentiation. Products generated by glycolysis are utilized in the pentose-phosphate pathway (PPP) and in lipid biogenesis; the latter was regulated by Acetyl-CoA carboxylase 1 (ACC1) but not by mitochondrial anaplerosis, as the cells have limited expression of Pyruvate dehydrogenase (PDH1). Anaplerosis is driven by amino acids, such as glutamine that is imported through the amino acid transporter SLC1A5 and metabolized by glutaminase 1 (GLS1). The metabolite 2-Hydroxyglutarate (2-HG), generated by Glutamic-oxaloacetic transaminase 1 (GOT1), suppresses the activity of the FOXP3 promoter. Other amino acid metabolites favor an epigenetic configuration that promotes the Th17-cell transcriptional program. **(B)** Deprivation of amino acids glutamine, serine, or methionine or ablation of GLS1 or of an enzyme involved in the one-carbon pathway Bifunctional methylenetetrahydrofolate dehydrogenase/cyclohydrolase (MTHFD2) or inhibition of the poly-amine pathway prevents Th17 cell proliferation. Increased ROS production, perhaps due to the lack of glutathione and substrate (succinate) for mitochondrial electron transport, alters epigenetic configuration to favor FOXP3 expression and Treg-mediated suppressive activities. **(C)** Pathogenic Th17 cells, signified by active mTORC1, lack of OXPHOS, production of IFNγ, and high ΔΨm, rely on the polyamine pathway and putrescin. **(D)** Ablation of mTORC1 and reduced ΔΨm result in Th17 cells expressing stemness markers T-cell factor 1 (TCF1) and Lymphoid enhancer-binding factor 1 (LEF1).

After exiting the thymus, naive T cells remain quiescent while circulating through blood and the lymphatics (30). The cells express low amino acid transporter levels, have low glucose transport capacity, and rely on OXPHOS and FAO (30). Expression of the T-bet gene, *TBX21*, an immune cell transcription factor in naive T cells, indicates that the cells are primed to become Th1 cells (39). This is reflected by chromosomal markers favoring a Th1 gene signature including activation of the *IFNG* gene locus (40). T-bet also appears to play a role in metabolism in these cells, as *Tbx21^−/−^* mice have increased visceral adiposity but are more insulin-sensitive, exhibiting reduced immune cell numbers and cytokine secretion specifically in the visceral fat depot, perhaps due to altered T-cell trafficking (14). The effector Th1 program is activated in lymph nodes (LNs) following TCR engagement. This leads to the upregulation of the glucose transporter GLUT1, activation of glycolysis and mTORC1, and differentiation of the cells (15). Th2 cells, in contrast, rely on mTORC2 for their differentiation that also inhibits the Th1 program (16, 41). nTregs and iTregs rely on OXPHOS and FAO during their resting state but require glycolysis and mTORC1 when activated in LNs or in the periphery (10, 42, 43). Perhaps paradoxically, however, this activation was also shown to reduce their suppressive activity (44). Tfh cells use glycolysis and OXPHOS selectively in response to changes in substrate availability in the GC where they reside (45). Being a focus of the review, Th17-cell metabolism will be described in more detail below. Interestingly, metabolism of activated Th17 cells has many similarities with that of Th1 cells. Antigen presentation in mesenteric LNs induces metabolic changes and the establishment of a Th17 differentiation program in naive T cells (46). Following TCR engagement, just as for Th1 cells, transcription of genes involved in glycolysis, including *GLUT1*, is upregulated (**Figure 1A**). Stromal interaction molecule 1 (STIM1) expressed in the ER undergoes conformational changes that augment Ca^2+^ release from intracellular compartments and its influx through cell membrane channels in Th1 and Th17 cells. These events promote glycolysis and OXPHOS and lead ultimately to IL-17 production, while RORγt level remains unaffected by the change in Ca^2+^ levels (47). Hence, not only is glycolysis, but also OXPHOS is essential for the early molecular events leading to Th17 differentiation. Induction of the transcription factors BATF and STAT3 are such early events (48). TCR engagement activates mTORC1 *via* PI3K/Akt, which also promotes cell differentiation (49). Th17 cells lacking Raptor lose the ability to develop effector functions (6). In addition, activated effector Th17 cells have a high ΔΨm (12). Interestingly, among T lymphocytes, Th17 cells are unique in displaying functional flexibility. For example, Th17 cells can transdifferentiate to both Tregs and pathogenic Th17 cells, the latter often associated with the ability to produce Interferon-γ (IFNγ) (**Figure 1C**) (1, 46). Th17 cells can transdifferentiate to pathogenic Th17 cells in response to severe bacterial and fungal infections (described in the section on *Changes in Th17-Cell Metabolism Are Related to Its Physiological Functions*) (46, 47).

## Th17-cell metabolism

Th17 cells, unlike Th1 cells, divert glycolysis-derived pyruvate away from the TCA cycle as PDH1 is downregulated in the cells (50). PDH1 is Ca^2+^ sensitive, but whether the increased levels of Ca^2+^ in Th17 cells in response to TCR stimulation influence PDH1 differently to Th1 cells has not been addressed (13). In Tregs, however, PDH1 is required for their function and, consequently, limiting autoimmunity, inflammation, and chronic disease (18). Since pyruvate is diverted from OXPHOS, Th17 cells will become dependent on amino acids as substrates for anaplerosis (**Figure 1A**). The importance of certain amino acids for Th17 functions is highlighted by the fact that only these cells among all T-cell subsets cease to proliferate when the glutamate-proline tRNA ligase EPRS1 is inhibited, or cysteine or methionine is depleted, resulting in phosphorylation of the stress sensor general control nonderepressible 2 (GCN2). The importance of glutamine as a substrate for Th17-cell metabolism is highlighted by that the glutamine transporter SLC1A5 and the glutamine-degrading enzyme glutaminase 1 (GLS1) are being expressed at high levels in these cells. Hence, the culture of Th17 cells in media lacking glutamine, or the ablation of GLS1, inhibits the function of the cells (15). Glutamate, the product of glutamine degradation, is a constituent to the reactive oxygen species (ROS)-scavenging glutathione but can also be metabolized to 2-hydroxyglutarate (2-HG) *via* α-ketoglutarate (α-KG) (51). GLS1 deficiency both increases ROS and alters cells’ epigenetic status by inducing trimethylation of histone H3K27 at many chromosomal locations (**Table 1**). Inhibition of ROS alters these epigenetic marks, indicating that glutamine supports Th17 cells by inhibiting ROS and its influence on epigenetic gene control. ROS is also an activator of mTORC1, underpinning the importance of this protein complex in Th17-cell functions. In support of this theme, ablation of the catalytic subunit of glutamate cysteine ligase (Gclc) in T cells results in glutathione depletion and impaired mTORC1 activity (52). Th1 cells, in contrast, adapt to GLS inhibition and increase glucose uptake for anaplerotic reactions to maintain cell phenotypes (15). Methionine restriction, in contrast, reduces the production of S-adenosyl methionine (SAM) that is required for histone H3K4 methylation at the promoter regions of Th17 cells, thus, inhibiting their proliferation and IL-17 production (**Figure 1B**, **Table 1**) (53).

Cytoplasmic acetyl-coenzyme carboxylase 1 (ACC1) is upregulated in Th17 cells. ACC1 utilizes pyruvate-derived acetyl-CoA as metabolite for lipid synthesis (54). Lipids generated because of this reaction interact with RORγt and increase its activity in driving Th17-cell differentiation. Acetyl-CoA produced from glutamine also regulates IL-17 production by acetylation of histone H3, thereby exposing the IL-17A promoter for RORγt transcriptional activity (17).

In addition, Th17 cells have a unique response pattern to environmental stresses. Thus, these cells cease to proliferate to deficit in specific amino acids (15, 55). In contrast, low O_2_ tension, both high and low levels of glucose, mannitol-induced osmotic stress, and high levels of NaCl promote Th17-cell proliferation (19, 56, 57). Low glucose levels reduce IL-2 production and STAT5 signaling, which is unfavorable for T cells other than Th17 cells (58). Relevant to these observations is that Th17 cells require a lower TCR signaling strength than Th1 cells to be activated. However, this results in reduced IL-2 production (58, 59). High levels of glucose, in contrast, induce Th17 cells. In this setting, neither glycolysis nor OXPHOS is affected, but high glucose levels induce mitochondrial ROS (mtROS) production. mtROS is released extracellularly where it converts TGFβ from its latent form to its active one that, in turn, supports the development of Th17 cells (57). Mannitol-induced osmotic stress promotes Ca^2+^ release from Th17 cells’ ER, and this influx augments Th17 cell responses (19). Compared with Th1 cells, Th17 cells are specifically regulated by HIF1α and mTORC1. Of note, in mice, HIF1α did directly associate with RORγt to promote Th17-cell differentiation (20). It is, thus, intriguing to speculate that this reliance of HIF1α reflects the Th17-cell localization to mucosal membranes where the O_2_ tension can be low and antigen-presenting cells are scarce (4, 22, 29).

As cited in the previous section, Th17 cells can transdifferentiate to Tregs or to pathogenic Th17 cells, and this process manifest metabolic alterations. Such alterations can be induced experimentally, or by disease. Experimentally induced metabolic dysregulation can induce Th17 transdifferentiation to Tregs. One carbon (1C) metabolism is important for T-cell activation (30). The 1C metabolism consists of a series of interlinking metabolic pathways, including the methionine and folate cycles. The methionine/folate pathways, thus, involve folate/methionine providing 1 methyl group for the synthesis of purine nucleotides for DNA synthesis, polyamines, amino acids, creatine, and phospholipids. One of the enzymes in the mitochondrial branch of the 1C pathway, the bifunctional methylenetetrahydrofolate dehydrogenase/cyclohydrolase 2 (MTHFD2), is important for Th17-cell functions. Inhibition of MTHFD2 results in reduced mTORC1 activity, increased OXPHOS, and reduced abundance of succinate and fumarate (60). MTHFD2 deficiency results in increased DNA demethylation including in the FOXP3 promoter-locus, thereby shifting pathogenic Th17 cells to acquire a Treg phenotype (**Table 1**) (60). Another study revealed that 2-HG produced by glutamate oxaloacetate transaminase 1 (GOT1) facilitated methylation and silencing of the FOXP3 promoter. Inhibition of GOT1 activity on the other hand converted Th17 cells to iTregs (51). Furthermore, chronic infection, malignancy, and T-cell exhaustion are known to upregulate the receptor PD-1 on T cells. PD-1 augmentation promotes FAO and inhibits glycolysis which could impede on Th17-cell effector function and promote a regulatory phenotype shift (61). Along similar lines, TGFβ and IL-6 upregulate CD73 expression on Th17 cells and this renders the cells anti-inflammatory in a tumor microenvironment (23). CD73 is an ecto-5’-nucleotidase that degrades AMP, derived from ATP or from NAD, generating the anti-inflammatory metabolite adenosine (23, 24). Adenosine, in turn, binds receptors on immune cells to trigger anti-inflammatory activities. Intriguingly, CD73 is also expressed on B cells (24).

Different approaches have been used to probe the heterogeneity of Th17 cells (62). Enrichment of Th17 cells based on their ΔΨm identified high-ΔΨm cells expressing higher levels of IL-17, while cells with low ΔΨm expressed higher levels of the stemness markers TCF1 and LEF1 (12). Furthermore, mice with T cells lacking Raptor produced Th17 cells with low metabolic activity, and expression of TCF1 (**Figure 1D**) (6). In contrast, Th17 cells with intact mTORC1 showed a capacity to transdifferentiate to pathogenic Th17 cells signified by the ability to produce IFNγ. A study using single-cell RNA sequencing (scRNA-seq) confirmed the Th17 cells’ metabolic heterogeneity. The study revealed that protective Th17 cells accumulate arginine while pathogenic Th17 cells synthesize and recycle polyamines, with putrescine being the mediator that best augments the cells’ activities. Inhibition of polyamine metabolism also promotes FOXP3 expression (**Figure 1C**) (62). In another study, putrescine was shown to have no impact on Th17 differentiation when added during *in vitro* cultures (63). These findings highlight the difference between studies of Th17-cell metabolism *in vitro* and *in vivo* and the value identifying of specific differences using scRNA-seq (62, 63).

## B-cell metabolism

**Figure 2:**
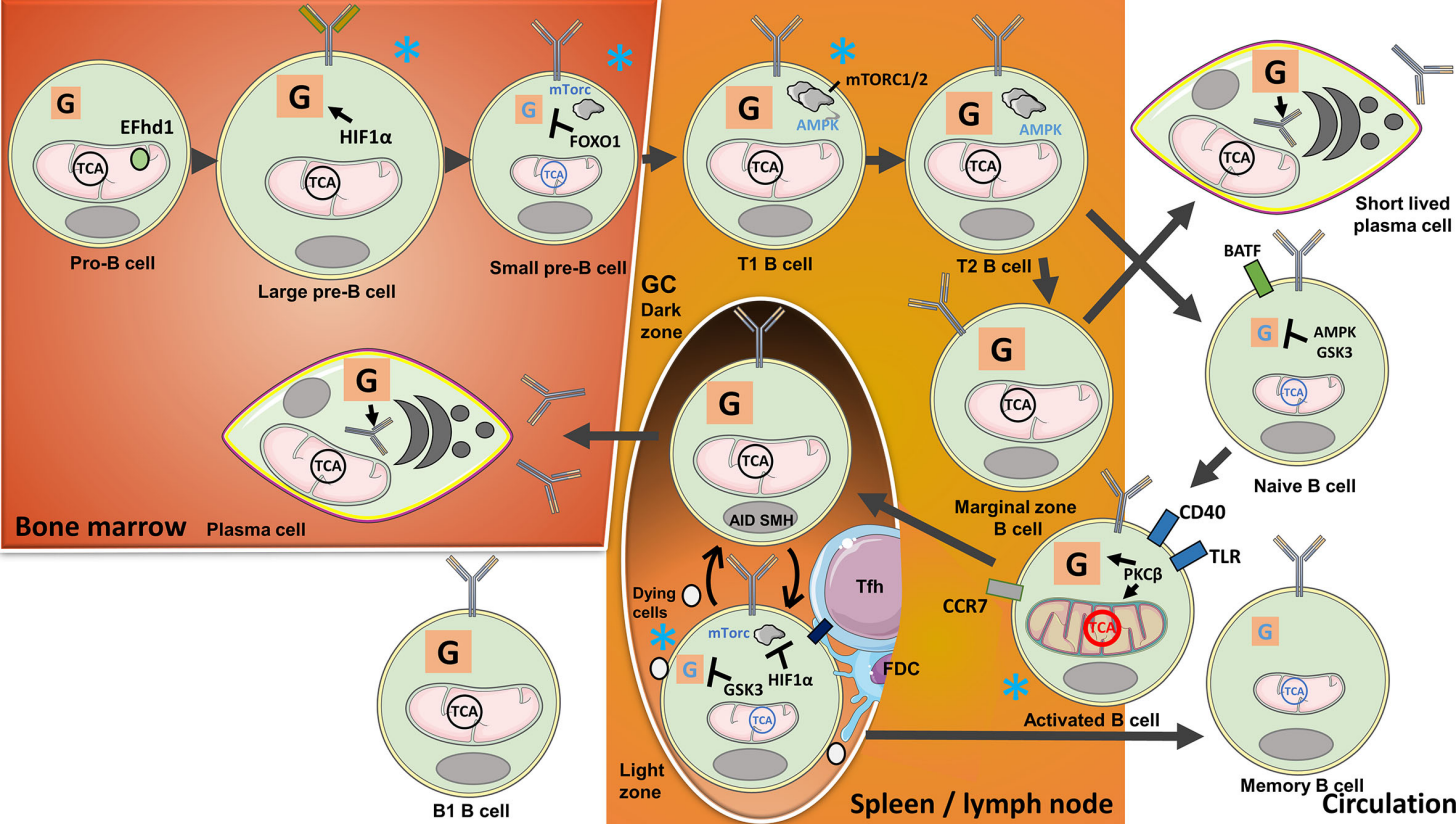
B-cell metabolism changes during their development, activation, and purging of clones with no available target antigens or those that are autoreactive. In the bone marrow, metabolism changes during B-cell development with the highest activity at pro-B cell and large B-cell stages: Heavy-chain gene rearrangements during the pro-B cell stage are associated with high ΔΨm, while cell expansion during the large B-cell stage is signified by increasing glycolytic activity. The mitochondrial Ca^2+^ binding EF-hand domain family member D1 (hEFd1) protects pro-B cells from heavy-chain synthesis-induced late surge in Ca^2+^. Large B cells localized to a niche with low O_2_ tension upregulate Hypoxia-inducible factor 1α (HIF1α) activating glycolysis during cell expansion. Light-chain gene arrangements and removal of self-reactive clones during the small pre-B-cell and immature B-cell stages are, in contrast, associated with suppressed mechanistic target of rapamycin 1(mTORC1) and low metabolic activity controlled by Forkhead box protein O1(FOXO1). Transitional (T) B cells again have high metabolic activity, OXPHOS, and mTORC1/2. Residual autoreactive clones are removed when the cells locate to the spleen and transition from T1 to T2 stage. Naive and Marginal zone (MZ) B cells develop from T2 B cells. Naive B cells found in the circulation have low metabolic activity regulated by AMP-activated protein kinase (AMPK) and Glycogen synthase kinase-3 (GSK3). Tonic B cell receptor (BCR) signaling and B-cell activating factor (BAFF) receptor engagement provide survival signals. When naive B cells encounter antigens, the cells are activated and undergo metabolic changes with the expansion of mitochondria guided by Protein kinase C β (PKCβ). Activated B cells will migrate to lymph nodes and the spleen, requiring T-cell help or Toll like receptor (TLR) stimulation for survival. T cell-stimulated B cells locate to B-cell follicles to initiate Germinal centre (GC) reactions. Highly metabolically active B cells proliferate in the Dark zone (DZ) and undergo activation-induced cytidine deaminase (AID)-guided somatic hypermutations (SHM). The cells subsequently move to the Light zone (LZ), where HIF1α, induced by low O_2_ tension, suppresses mTORC1, while GSK3 suppresses glycolysis. The cells compete for selection by follicular T cells (Tfh) cells and follicular dendritic cells (FDCs) in the LZ. Selected cells utilize lipids from unselected counterparts for anaplerosis. The cells can reenter the DZ for further selection, or leave the GC, in the latter case either differentiating to long-lived plasma cells (LLPC) or memory B cells. LLPCs with high metabolic activity will locate to the bone marrow, there producing antibodies. Glycolysis provides a substrate for glycosylation of antibodies. Circulating memory B cells, on the other hand, are signified by low metabolic activity. MZ B cells, another progeny of transitional B cells, develop into short-lived plasma cells with a metabolic signature similar to LLPCs, independently of GC reactions. B1 cells developing in the embryo are signified by high glycolysis and OXPHOS. Blue stars indicate B-cell stages where clones with low antigen specificity or self-reactivity are excluded.

Early studies have identified two distinct B-cell lineages in mice and, probably in humans. These two B-cell lineages are distinguished phenotypically by the expression of CD5, a primarily T cell-associated membrane protein. The two subsets were designated conventional or CD5^-^ B cells (also called B2 cells) and CD5^+^ B cells (B1 cells) that produce natural polyreactive IgM antibodies. The developmental pathway of the B2-cell lineage is shown in **Figure 2**. Cellular metabolism during clonal B2-cell selection/exclusion influenced by their BCR is shown in **Table 2** and **Figure 2**. B1 cells have been suggested to have diverged during the embryonic stage in mice and have the ability for self-renewal. These cells are endowed with high glycolysis and OXPHOS activity (64). In addition to these two lineage cells, a subset of B cells that regulates immunity and inflammation and promotes differentiation of naive T cells to iTregs, called Bregs, have been identified. Bregs’ functions are contact-dependent and can involve IL-10 production [metabolism reviewed in Iperi et al. (65)].

Conventional B2 cells are produced in the bone marrow from hematopoietic stem cells. B2 precursors undergo gene rearrangements in the bone marrow that ultimately result in the expression of diverse BCR repertoires at the latter stages of pro- and pre-B-cell developments. The progenitor cells localize to different niches in the bone marrow with varying degrees of oxygen tension, thus, influencing the cells’ metabolic characteristics. Pro-B cells have the highest Δψm, but this is incrementally diminished during the subsequent stages of their development. OXPHOS activity is similar in pro- and large pre-B-cell stages, but the latter cells have the highest glycolytic activities among the progenitors, accompanied by high levels of ROS. Reflecting a niche with low oxygen tension, HIF1α is active during the pre-B-cell stages, thus, driving glycolysis. When HIF1α is experimentally deleted, B cells switch their metabolism to TCA anaplerosis for their energy needs (66). The Ca^2+^-binding protein Swiprosin-2/EF-hand domain family member D1 (EFhd1) was found to be located to the inner mitochondrial membrane of pro-B cells. Due to the emergence of the pre-BCR in late pro-B cells, Ca^2+^ is directed from the ER to the mitochondria. This results in an increased mitochondrial pH and a drop in Δψm and ATP production. Detectable “mitoflashes,” likely due to a drop in mitochondrial pH, occur in regions with EFhd1 and Ca^2+^. This is likely to be a means to rescue Δψm and increase ATP production in these cells. Ca^2+^ signaling downstream of SYK is important for pre-BCR signaling in pro-B cells and is related to these noted events (27, 67). EFhd1 is downregulated at the pre-B-cell stage consequent to the localization of the pre-BCR to the membrane and the migration of the cells to a niche with low O_2_. Subsequent anaplerotic reactions provide protection from ROS and growth (67, 68). Glycolysis and OXPHOS are reduced in pre-B cells compared with the B-cells at their earlier developmental stages. Downregulation of PI3K/Akt and ROS-mediated induction of FOXO1 together with the expression of the lineage-defining transcription factor paired box 5 (PAX5) induce cell cycle arrest while light-chain genes are rearranged (69). Provided that the BCR does not bind avidly to self-antigens, a program of maturation follows surface expression of the complete BCR (25).

Immature B cells eventually exit the bone marrow as transitional 1 (T1) B cells. T1 B cells express higher levels of genes involved in ribosome biogenesis, aerobic respiration, and mTORC1 than B cells at later stages of their maturational pathway but, in contrast, express low AMPK levels. Interestingly, studies of mice deficient in the mTORC2 component Sin1 showed a reduction in T1 and T2 B cells. mTORC2 was reported to increase Akt signaling, stabilize mTORC1, and suppress glycogen synthase kinase-3 (GSK3) (9). *In vitro* treatment with the AMPK agonist 5-Aminoimidazole-4-carboxamide ribonucleotide (AICAR) supports the evolution of T1 B cells to conventional B2 cells (7).

*In vivo*, the progression of T1 to T2 B cells takes place mostly in the spleen where tolerance checkpoints are in place to eliminate residual autoreactive T1 B-cell clones and to prevent their transition to mature B cells (26). B cells that survive peripheral tolerance checkpoints develop either to mature naive B cells or to marginal zone B cells (MZB, see below). Naive B cells are metabolically inactive and circulate through the blood and lymphatics (30). Tonic signaling through the BCR and signals mediated by B-cell activating factor (BAFF) preserve homeostatic mitochondrial signals in naive B cells (70). In contrast, AMPK, GSK3, and PAX5 suppress glucose uptake and maintain quiescence in mature non-activated B cells (7, 71, 72).

Mature naive B cells are activated when they encounter and bind their target antigens through their BCRs, mostly, in the presence of T-cell help. This results in Myc- and PI3K-mediated GLUT1 expression, glycolysis, and glutamine-supplemented anaplerosis for OXPHOS (73). The cells initiate a transcriptional program to remodel the mitochondria and increase their glycolytic activities. To prevent abnormal activation and/or the expansion of residual uncensored autoreactive B cells, the cells require a second stimulation within 24 h that can either be T cell mediated (CD40) or through Toll-like receptor (TLR9) (74). Stimulated B cells upregulate chemokine (C-C motif) receptor 7 (CCR7) and move to nearby secondary lymphoid organs to proliferate inside or outside GCs. Ectopic GCs can be initiated in non-lymphoid organs during chronic inflammation and autoimmunity. Without a second activation signal, increasing intracellular Ca^2+^ levels transported through calcium channels and increasing levels of ROS will ultimately lead to the apoptosis of antigen-primed B cells (74).

In lymphoid organs, on the boundaries between T- and B-cell areas, B cells present fragments of the antigen recognized by their BCRs for cognate Th-cell interaction together with costimulatory signals. These B cells can develop independent of GCs into short-lived extrafollicular plasma cells that are important for the initial wave of protective antibodies. Alternatively, these cells can differentiate to unswitched memory B cells. Other B cells will migrate into B-cell follicles to initiate GC reactions and become founders for clones whose antibodies acquire increasing affinity for the antigen in the newly formed GCs. GCs will evolve into two zones, the dark zone (DZ) closest to the T-cell zone in LNs and the LZ that is closest to the capsule in LNs and marginal zones of the spleen and with cells shuttling in-between guided by chemokine signals. B cells actively divide in the DZ and express activation-induced cytidine deaminase (AID) enzyme and mediate somatic hypermutation (SHM). BCR and CD40 are required for Myc-regulated expression of metabolic enzymes and membrane transporters. B cells do not proliferate in the LZ and, instead, compete for selection during interactions with antigens expressed on follicular dendritic cells (FDCs) and obtaining help from Tfh cells. FDCs and Tfh cells regulate positive selection, while Tfr cells suppress the output of activated B cells (28). Strength of the B cell/Tfh cell interaction determines later proliferation efficiency in the DZ (75). As the LZ is farthest from the blood supply and oxygen in GCs, HIF1α is activated. This activation prevents AID activity and, thereby, restricts Ig class switching but, interestingly, not SHM. mTORC1 regulates HIF1α and the Von Hippel–Lindau tumor suppressor while GSK3 protects B cells from deprivation of glucose and nutrients (5, 8, 71). Fatty acids (FAs) from other surrounding B cells, dying due to lack of costimulation, supply OXPHOS (76). PKCβ regulates antigen presentation in B cells and, therefore, the development of Tfh cells to support further B-cell proliferation and differentiation (77). Class-switched B cells subsequently undergo repeated expansion in the DZ or exit the GC. Memory B cells and long-lived plasma cells (LLPCs) have exit cues from the GC that correlate with BCR affinity and time since the response began (75). Memory B cells circulate and when reactivated by their target antigens start producing antibodies or initiate another GC reaction for further affinity maturation. Memory B cells are signified by low-level metabolism relying primarily on OXPHOS. LLPCs migrate to the bone marrow and, guided by the transcription factor Blimp-1, mature to cells dedicated to antibody production. These cells have high OXPHOS activity and glycolysis, the latter providing the substrate for glycosylation of the antibodies that are produced (78).

Activated B cells residing in the MZ of lymphoid organs express TLRs and are activated through TLR9 for survival (79). These cells act as an early response element and produce, mostly polyreactive, antibodies with low affinity. Stimulation through the TLR together with transmembrane activator and CAML interactor (TACI) activates mTORC1 signaling in MZB cells leading to high expression of the glucose transporter GLUT1 and consumption of glucose. This leads to B-cell proliferation and, subsequently, to immunoglobulin G (IgG) class switching and differentiation to plasmablasts (80). IL-10 production by MZ precursor B cells has been shown to regulate the differentiation of Th17, Tfh, and Tfr cells (81). The MZ precursors could therefore, be considered to have potential Breg-related functions (65).

## Changes in Th17-cell metabolism are related to its physiological functions

During homeostasis, IL-22-producing Th17 cells are primarily found in the mucosa of the intestine conferring protection and supporting intestinal barriers. DCs present antigens from the intestinal microflora to naive T cells in mesenteric LNs leading to the expression of RORγt and the gut homing receptor α4β7 by the cells. The cells subsequently proliferate and migrate to Peyer’s patches and intestinal mucosa to start protective activities (21, 46, 82). Infection with the commensal segmented filamentous bacteria (SFB) in mice results in effector Th17 cells in the intestine with elongated mitochondria, relying on OXPHOS to produce Nicotinamide adenine dinucleotide phosphate (NADPH) and glutathione. Infection with the pathogen *Citrobacter rodentium*, in contrast, results in pathogenic Th17 cells that coproduce IL-17 and IFNγ. The latter Th17 cells have fragmented mitochondrial morphology and rely on glycolysis for their metabolic needs. The IFNγ-producing Th17 cells cannot transdifferentiate from resident intestinal Th17 cells but are originated in the LNs. The pathogenic Th17 cells, unlike Th17 cells from SFB-infected mice, can also disseminate to spleens of infected mice (46). The bacterial flora produces short-chain fatty acids (SCFAs) such as pentanoate. These SCFAs act in Th17 cells as a histone deacetylase inhibitor, thereby suppressing IL-17 transcription. Pentanoate from SFB can, therefore, reduce Th17 activity in the intestine (83).

Studies in mice have revealed that Th17 cells differentiate to tissue-resident memory T (Trm) cells to provide protection against bacterial and fungal infections in the lungs and skin (84, 85). These cells provide protection mainly through the recruitment of neutrophils (47). Deletion, or mutations described in patients, of *STIM1* that regulates Ca^2+^ uptake in Th17 cells leads to impaired fungal defenses, dissemination in kidneys, and eventual death. *In vitro* analyses of non-pathogenic and pathogenic STIM1-deficient Th17 cells showed that the non-pathogenic subset of cells was most dependent on STIM1. Expression of HIF1α target genes, mTORC1 activation, glycolysis, and OXPHOS are reduced in these cells, while the FOXO1 pathway is upregulated. Thus, specifically for non-pathogenic Th17 cells, extracellular Ca^2+^ is important for effective immunity to fungal infections (47). Hence, non-pathogenic Th17 cells rely on Ca^2+^ imported *via* STIM1, glycolysis, and OXPHOS, while pathogenic Th17 cells, stimulated by severe bacterial and fungal infections, utilize glycolysis only (46, 47). Low oxygen tension (activation of HIF1α) may thus, promote a pathogenic phenotype. Such findings are in line with a recent study comparing pathogenic and non-pathogenic Th17 cells in a model of multiple sclerosis (22). This, apparently, is associated with a lack or reduced supply of amino acids (15, 49).

## The contribution of dysregulated Th17-cell metabolism to disease pathogenesis

### Th17-cell metabolism in psoriasis

**Figure 3:**
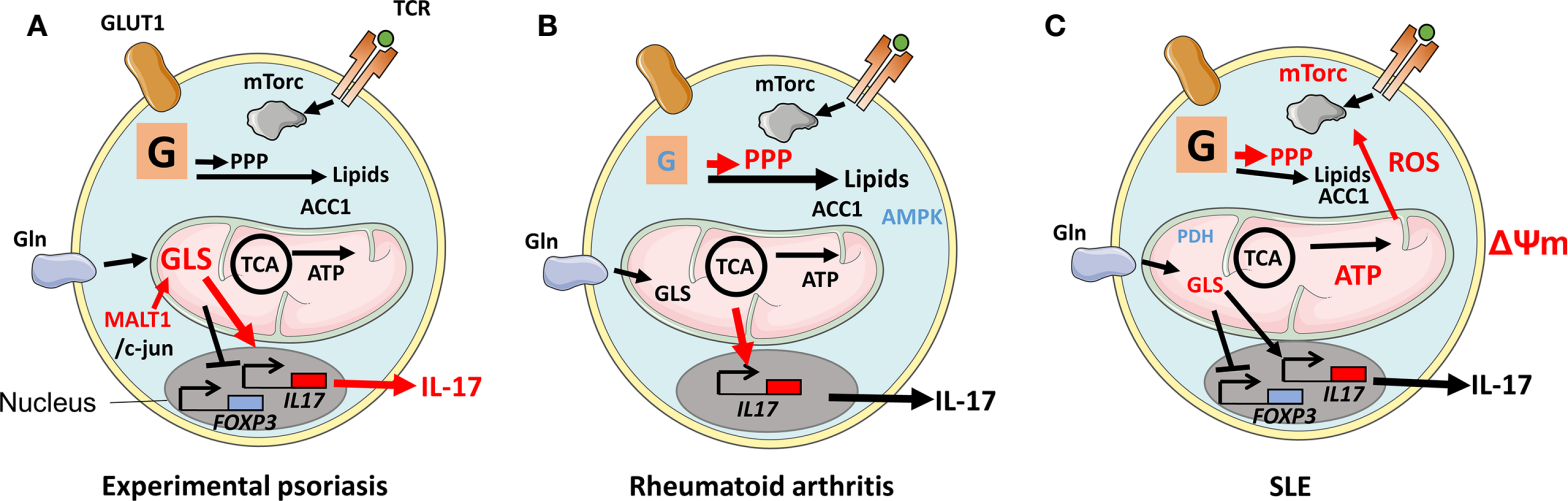
Disease-associated changes in metabolic pathways in Th17 cells. **(A)** During experimental psoriasis, dysregulated expression of Mucosa-associated lymphoid tissue lymphoma translocation protein 1 (MALT1) results in overexpression of Glutaminase 1 (GLS1) and generation of acetyl-CoA from metabolized glutamine. Histone H3K9Ac and H3K27Ac acetylation marks were found in the *il17* gene promoter leading to increased IL-17 production and psoriasis. **(B)** Defective glycolysis and TCA cycle in naive T cells in RA patients and a lack of mechanistic target of rapamycin (mTORC1) restraint due to inactive AMP-activated protein kinase (AMPK) result in an inflammatory phenotype with increased migratory properties and the production of IL-17 and IFNγ. The metabolism of glutamine in Th17 cells contributes to a Th17 cell phenotype. **(C)** Th17 cells in patients with SLE are signified by increased glycolysis and OXPHOS, elongated mitochondria, elevated ΔΨm, and mitochondrial reactive oxygen species (mtROS). The elongation of mitochondria is a consequence of oxidative stress-induced reduction of Rab4A-mediated recycling of Dynamin-related protein 1 (Drp1). Rab4A defect also results in increased mTORC1 activity. Reduced expression of Protein phosphatase 2 (PP2A) results in reduced expression of Pyruvate dehyrogenase 1 (PDH1).

Psoriasis is the consequence of uncontrolled proliferation of dermal keratinocytes. The disease affects 2%–3% of populations worldwide and manifests in scaly plaques that in the disease's severe from cover more than 10% of the body. Th17 cells, present in the dermis of patients as IL-17-producing Trm cells, contribute to psoriasis pathology, evidenced by therapeutic efficacy of treatment with anti-IL-17 antibody in reducing plaques (86). Th17-cell metabolism was studied in an animal model of psoriasis and discovered that the production of the mucosa-associated lymphoid tissue lymphoma translocation protein 1 (MALT1) was dysregulated in Th17 cells. MALT1 stabilizes c-jun that could then bind and activate GLS1 expression, resulting in increased glutaminolysis in Th17 cells. GLS1’s overexpression in patients’ Th17 cells leads to high levels of acetyl-CoA production. This metabolite, in turn, induces histone H3 acetylation; specifically, H3K9Ac and H3K27Ac marks in the IL-17A gene promoter region resulting in increased IL-17 production leading to pathology (**Figure 3A**). This investigation revealed that GLS, or MALT1 inhibitors, previously considered as cancer therapy, can be a potential treatment for psoriasis (17).

### Th17-cell metabolism in rheumatoid arthritis

Rheumatoid arthritis (RA) is a debilitating disease mainly affecting joints in 0.5%–1% of populations worldwide. The synovial lining of RA joints is targeted by an immune response that causes juxta‐articular and generalized bone loss. The level of IL‐17 is reduced at disease onset compared with before onset (87). Naive CD4+ T cells in patients with established RA are, however, prone to differentiate to Th17 cells (39). Naive T cells from RA patients manifest defective glycolysis resulting in diminished levels of ATP, low ROS, and an overall defective mitochondrial function (88). The GDP-forming β subunit of succinate-CoA ligase (SUCLG2), part of the TCA, was reported suppressed, resulting in reversal of the cycle. This reversal induces IL-17- and IFNγ-mediated inflammation, as shown by the transfer of patient peripheral blood mononucleated cells (PBMCs) to immune compromised NOD Scid gamma mice that harbor patient synovium transplants. Overexpression of SUCLG2 in these PBMCs reduced IL-17 production. As a consequence of the SUCLG2 defect, citrate was transported out of mitochondria and converted to acetyl-CoA, clustering mitochondria perinuclear and increasing T-cell invasiveness (31). Inflammation and migratory properties suggest that mTORC1 activation and efficient OXPHOS are involved. However, the above-cited studies suggest that these pathways are dysregulated in RA. AMPK was found to be displaced and, thereby, could not regulate mTORC1, resulting in high-level production of IL-17 and IFNγ. This AMPK defect could, therefore, explain the inflammation caused by the cells (**Figure 3B**) (11). Indeed, an appropriately located AMPK is able to drive FAO favoring Tregs instead of Th17 cells (10). In arthritic joints, there is an enrichment of Th17 cells that can promote arthritis by inducing the production of pro‐inflammatory cytokines and receptor activator of nuclear factor κB ligand (RANKL) while inhibiting apoptosis in synoviocytes. Hypoxia in the inflamed synovium favors both the development of Th17 cells and T cells with defective OXPHOS. Furthermore, levels of glutamine and glutamate are high in the synovial fluid of RA patients (89). In a study using an animal model of RA, mTORC1 and glutamine metabolism were simultaneously suppressed using rapamycin and 6-diazo-5-oxo-L-norleucine (DON). This combination reduced Th17 proliferation and arthritic scores. Rapamycin, but not DON, also increased Tregs. Although prior usage of DON as therapy for malignancy was hampered by side effects, inhibition of autoreactive immune cells’ utility of glutamine might be worth considering (90, 91). The inflammatory environment increases the level of lactate in patients’ synovial fluid. One study observed that Th17 cells that took up lactate were unable to migrate, therefore, residing in the synovium (92). It should be pointed out, however, that although Th17 cells are a prominent part of RA pathology, treatment using anti-IL-17 has not shown strong beneficial effects at a level comparable to anti-TNFα (93). A better characterization of these heterogeneous cells and stratification of patients can, potentially, provide better understanding of when anti-IL-17 therapy would be of optimal therapeutic benefit. In that context, a recent study has shown that T cells from RA patients, already as naive, have a dysregulated malate-aspartate cycle, resulting in elevated levels of NAD^+^ and the expansion of the ER where TNFα was produced. T cells were noted to be a major producer of TNFα compared with monocytes/macrophages (94, 95). Anti-TNFα is currently used as an efficacious therapy in RA patients, but the targeting of the mitochondria-ER cross talk might be a novel more specific therapeutic option (94). Studies have shown that non-responsiveness to anti-TNFα is, however, associated with a Th17-cell signature, potentially, indicating that T cells from these individuals suffer from a metabolic disorder that is distinct from anti-TNFα responder patients (96). Better understanding of such disease-associated defective metabolism might prove useful for the development of therapeutics to induce durable tolerance in anti-TNFα non-responders (60, 62).

### Th17-cell metabolism in systemic lupus erythematosus

Systemic lupus erythematosus (SLE) is another autoimmune disease that affects 20–70 individuals per 100,000 of the population. Patients suffer a range of symptoms; skin and kidneys are affected, probably, due to a defective removal of apoptotic bodies leading to the accumulation of cell debris of nuclear, cytosolic, and membrane origins. This debris activates autoreactive B cells in GCs and elsewhere to proliferate and activate autoreactive T cells leading to the production of anti‐nuclear autoantibodies that form complement- and phagocyte-activating immune complexes. Th17 cells in SLE are signified by increased mTORC1, glycolysis, and OXPHOS. Mitochondria from lymphocytes in SLE patients are elongated, have high ΔΨm, and produce mtROS (97). Excessive elongation of mitochondria can be explained by increased degradation of Drp1, which regulates mitochondrial fission. High levels of oxidative stress lead to overexpression of the regulator of endocytic recycling Rab4A. Rab4A, which is genetically associated with SLE, therefore, recycles Drp1 leading to the elongated mitochondria phenotype (32). A long terminal repeat polymorphism in the *RAB4A* promoter has, moreover, been identified and shown to be associated with SLE. This induces mTORC1 activity in T cells in the patients (98). The protein phosphatase 2A (PP2A) is another protein genetically associated with SLE and is shown to be reduced in Th17 cells in the patients. This finding is related to the PP2A ability to induce the expression of the Th17-inhibiting mitochondria protein PDH1 (**Figure 3C**) (33, 50). Th17 cells directly promote GC reactions, or produce IL-21 that can influence Tfh, and animal studies have indicated that the cells can transdifferentiate to Tfh cells (1, 34, 99). It is currently unknown whether the overactive Th17 cells associated with SLE can influence Tfh and Tfr present in GC and that are involved in the regulation of B-cell proliferation.

## Defective B-cell metabolism and role in chronic inflammatory diseases

B cells differentiate to plasma cells that produce antibodies conferring humoral immunity against infectious pathogens and to memory cells. As cited in the B-cell section, metabolism is continuously regulated and changes selectively during B-cell development and activation, so they can develop a highly variable antibody repertoire that does not react with self (**Figure 2**, **Table 2**). In addition to phases of B-cell development, factors such as severity and chronicity of an infection and the age of an individual also influence B cells’ metabolic properties. Aberrant regulation of cellular metabolism in B cells can lead to chronic inflammation and autoimmunity (100–102).

In RA and SLE, it is widely recognized that a breach in B-cell tolerance is an initiating step for disease development (103). In RA, B cells produce autoantibodies that recognize citrullinated antigens (anti-CCP) or the Fc part of IgG [rheumatoid factors (RFs)], the presence of which is associated with a more severe disease (104). In SLE, autoantibodies are mostly specific for nuclear antigens. Research is ongoing to understand how alteration to the B-cell metabolism in these diseases leads to immune dysregulation and autoimmunity. Assessment of the B-cell subset present in synovial tissues and synovial fluids has identified switched memory and double-negative memory B cells. These cells produce inflammatory cytokines when cultured in hypoxic conditions that mimic the synovial microenvironment. A proportion of these cells express PD1 and have active mTORC1 and a glycolytic gene signature. Assessment of intracellular NAD^+^ levels confirmed that the cells are primarily driven by glycolysis (105). As cited earlier, active glycolysis is a feature of activated antibody-producing B cells.

In SLE, B-cell metabolic dysregulation can take place during the GC reaction and in B cells that produce antibodies independent of the GC reaction. One study noted increased mTORC1 activity in B cells from patients with SLE, and the presence of cells with this activity correlated to plasmablast accumulation (106). Unswitched memory B cells develop independently of GC reactions (107). The study showed that *in vitro* stimulation of unswitched memory B cells with CpG/TLR9 and IFNα led to the development of plasmablasts dependent on mTORC1. CpG-only stimulation of the unswitched memory cells on the other hand induced a memory cell phenotype that was promoted by AMPK augmentation (106). Specific stimulation and mTORC1 upregulation can, therefore, promote B-cell pathology independent of GCs. The potential involvement of GC reactions in SLE pathology has, on the other hand, been proposed based on observations that autoantibodies in the disease are generated because of defects in the clearance of apoptotic cells within the LZ of GCs leading to BCR-mediated internalization of nuclear antigens and TLR-mediated activation. B cells that undergo GC reactions express high levels of the anti-inflammatory adenosine generating ecto-5’-nucleotidase CD73. A study noted inefficient functions of B cells from patients due to defective CD73’s nucleotidase activity (24). The authors suggested that the CD73 defect may promote autoimmunity due to two factors. First, there is the lack of adenosine that can render Th cells to differentiate to pro-inflammatory cells. This enables the Th cells to promote the survival of autoreactive B cells. Second, the accumulation of undegraded AMP prompts inflammation through augmenting IL-6 production by B cells (24). In GCs, mTORC1 activity is required for the selection of B cells in the LZ (8). A study using the Roquin lupus mouse model reported that treatment of mice with metformin reduced the number of GC B cells. Furthermore, Tfh and Th17 cells were inhibited, while Tregs were increased by treatment with metformin. Inhibition of immune cells was associated with an increase in AMPK levels (108). The study, therefore, concluded that therapeutic targeting of metabolic pathways might be a strategy to suppress autoimmune GC reactions. Th17 cells may also be involved, as they play an important role in the GC reactions (34). The interaction between B cells and Th17 cells changes the metabolism of Th17 cells and leads to their activation. After TCR engagement, naive B cells prompt IL-17 production by Th17 cells through the inducible T-cell costimulator (ICOS)/inducible T-cell costimulator ligand (ICOSL) interaction (94). Intriguingly, naive B cells appear to be better inducers of T-cell responses than memory B cells. This interaction induces mTORC1 and glycolysis in the Th17 cells and IFNγ production (36, 109). T cells in patients with RA and SLE express ICOS, and the level appears to be associated with RA patients’ plasma anti-CCP and RF levels. In line with the dysregulated Th17-cell phenotypes described earlier, naive B cells induce higher levels of IL-17 in RA and SLE patients’ Th17 cells than Th17 cells from healthy controls (36).

## Conclusions and prospects

Metabolic activity is intertwined with the functional status and stage of development and activation of immune cells and is impacted by disease. Th17 cells differentiate in response to various stresses but they do not utilize pyruvate for anaplerosis. Thus, while protective Th17 cells are reliant on glycolysis and OXPHOS, pathogenic Th17 cells only utilize glycolysis and produce putrescine that may reflect the environment in which they reside. Th17 cells are vulnerable to deficiency of certain amino acids or to deregulation of their processing in mitochondria. Stress increases Δψm, leading to ROS production, that would be sensed by mTORC1, and this will favor pathogenic Th17 transdifferentiation during low oxygen tension. Furthermore, deprivation from amino acids reduces survival but also influences epigenetic regulation to favor Th17 to Treg transdifferentiation. The tumor microenvironment can promote such transdifferentiation. Further studies are required to understand the physiological relevance of such transdifferentiation and whether this has a role in protection from disease or a break of tolerance. Disturbances in the transcription of genes involved in metabolism can render Th17 cells hyperactive in patients with psoriasis, RA, and SLE. Furthermore, inherited genetic changes can contribute to immune hyperreactivity. It remains to be determined, however, whether such hyperactivity directly influences/promotes the expansion of B-cell autoreactivity too, directly or indirectly Th17 cells.

During their maturation, B cells are censored at several checkpoints. At each of these developmental stages, B cells manifest different metabolic signatures. The activity of mTORC1/2 in B cells is associated with effector functions and antibody production, while AMPK promotes B cells to become memory cells. Furthermore, unlike Th17 cells, B cells display a metabolic plasticity and can replenish anaplerosis through glycolysis. Recent studies have revealed that metabolism-associated activation, stress, and exhaustion can promote B cell-mediated pathology through the breach of their tolerance. Such aberrant metabolism seems to influence both B cells involved in GC reactions and the ones that mature extrafollicularly. Furthermore, Bregs affected by aberrant metabolism lack suppressive abilities leading, potentially, to autoimmune pathology. However, knowledge on how altered metabolism influences B-cell tolerance at epigenetic levels is currently lacking.

Although Th17 cells and B cells are part of different lineages, there are similarities with their cellular metabolic responses due to similarities in microenvironments in which they can reside and are activated. In addition, disease-associated polymorphisms are known to alter metabolism in both cell types leading to the breach to immunological tolerance and the transdifferentiation of Th17 to pathogenic cells. Furthermore, Th17 cells contribute to GC reactions and the production of autoantibodies by contribution to B-cell differentiation to plasma cells. However, the metabolic status of B cells and/or Th17 cells during their interactions, leading to disease, has not yet been thoroughly examined (34, 35). Bregs, on the other hand, regulate Th17 cells. Better understanding of metabolic properties of the distinct functional subsets of B cells and T cells and how they influence each other can be of profound importance in understanding the pathogenesis of inflammatory diseases. Such information can potentially lead to the discovery of new therapeutic strategies. As overviewed in this review, such therapeutic strategies could involve inhibition of glutaminolysis, or supplementation with the SCFA pentanoate to alter cell metabolism. Future studies may need to define at what cell stage/type is modulation of metabolism most beneficial for disease amelioration while retaining the immune system intact and functional.

## Author contributions

JB, TT and RM wrote the first draft which was critically scrutinized by SH and DG. RM finalized the manuscript which was then approved by all the co-authors.

## Conflict of interest

The authors declare that the research was conducted in the absence of any commercial or financial relationships that could be construed as a potential conflict of interest.

## Publisher’s note

All claims expressed in this article are solely those of the authors and do not necessarily represent those of their affiliated organizations, or those of the publisher, the editors and the reviewers. Any product that may be evaluated in this article, or claim that may be made by its manufacturer, is not guaranteed or endorsed by the publisher.

## Glossary

**Table:**

2-HG	2-hydroxyglutarate
ACC1	acetyl-coenzyme carboxylase 1
acetyl-CoA	acetyl-coenzyme A
AID	activation-induced cytidine deaminase
APC	antigen-presenting cell
anti-CCP	anti-cyclic citrullinated peptide antibody
ATP	adenosine triphosphate
Akt	Ak strain transforming (also known as protein kinase B or PKB)
AICAR	5-Aminoimidazole-4-carboxamide ribonucleotide
AMPK	AMP-activated protein kinase
BATF	basic leucine zipper ATF-like transcription factor
Bcl-6	B-cell lymphoma 6
BCR	B-cell receptor
Breg	regulatory B cell
CCR7	chemokine (C-C motif) receptor 7
ΔΨm	mitochondrial membrane potential
DC	dendritic cell
DON	6-diazo-5-oxo-L-norleucine
Drp1	dynamin-related protein 1
EFhd1	EF-hand domain family member D1
ETC	electron transport chain
FAO	fatty acid oxidation
FDC	follicular dendritic cell
FOXO1	forkhead box protein O1
FOXP3	forkhead box P3
GATA-3	GATA-binding protein 3
GCN2	general control nonderepressible 2
GC	germinal center
GLS1	glutaminase 1
GLUT1	glucose transporter 1
GOT1	glutamate oxaloacetate transaminase 1
GSK3	glycogen synthase kinase-3
HIF1α	hypoxia-inducible factor 1α
ICOS	inducible T-cell costimulator
ICOSL	inducible T-cell costimulator ligand
LEF1	lymphoid enhancer-binding factor 1
LLPC	long-lived plasma cell
LN	lymph node
LZ	light zone
MALT1	mucosa-associated lymphoid tissue lymphoma translocation protein 1
MTHFD2	bifunctional methylenetetrahydrofolate dehydrogenase/cyclohydrolase
mTORC	mechanistic target of rapamycin
mtROS	mitochondrial reactive oxygen species
MZB	marginal zone B cell
NADH	nicotinamide adenine dinucleotide
NADPH	Nicotinamide adenine dinucleotide phosphate
OXPHOS	oxidative phosphorylation
PAX5	paired box 5
PDH1	pyruvate dehydrogenase 1
PI3K	phosphatidylinositol 3-kinase
PKCβ	protein kinase Cβ
PP2A	protein phosphatase 2A
PPP	pentose-phosphate pathway
Rab4A	Ras-related protein Rab-4A
RANKL	receptor activator of nuclear factor kappa-β ligand
RF	rheumatoid factor
RORγt	RAR-related orphan receptor γt
ROS	reactive oxygen species
SAM	S-adenosylmethionine
SCFAs	short-chain fatty acids
SFB	segmented filamentous bacteria
SHM	somatic hypermutation
SUCLG2	GDP-forming β subunit of succinate-CoA ligase
STAT3	signal transducer and activator of transcription 3
STIM1	stromal interaction molecule 1
SYK	spleen-associated tyrosine kinase
T1/2 B cell	transitional 1/2 B cell
TACI	transmembrane activator and CAML interactor
T-bet	T-box expressed in T cells
TCA	tricarboxylic acid
TCF1	T-cell factor 1
TCR	T-cell receptor
Tfh	follicular helper T cell
Tfr	follicular regulatory T cell
TGFβ	transforming growth factor-β
TLR	Toll-like receptor
Treg	regulatory T cell
Trm	tissue-resident memory T cell

## References

[1] Stockinger B Omenetti S . The dichotomous nature of T helper 17 cells. Nat Rev Immunol (2017) 17(9):535–44. doi: 10.1038/nri.2017.50 28555673

[2] Chung Y Tanaka S Chu F Nurieva RI Martinez GJ Rawal S . Follicular regulatory T cells expressing Foxp3 and bcl-6 suppress germinal center reactions. Nat Med (2011) 17(8):983–8. doi: 10.1038/nm.2426 PMC3151340 21785430

[3] Stadhouders R Lubberts E Hendriks RW . A cellular and molecular view of T helper 17 cell plasticity in autoimmunity. J Autoimmun (2018) 87:1–15. doi: 10.1016/j.jaut.2017.12.007 29275836

[4] Konjar S Pavsic M Veldhoen M . Regulation of oxygen homeostasis at the intestinal epithelial barrier site. Int J Mol Sci (2021) 22(17):9170. doi: 10.3390/ijms22179170 34502078 PMC8431628

[5] Cho SH Raybuck AL Stengel K Wei M Beck TC Volanakis E . Germinal centre hypoxia and regulation of antibody qualities by a hypoxia response system. Nature (2016) 537(7619):234–8. doi: 10.1038/nature19334 PMC5161594 27501247

[6] Karmaus PWF Chen X Lim SA Herrada AA Nguyen TM Xu B . Metabolic heterogeneity underlies reciprocal fates of TH17 cell stemness and plasticity. Nature (2019) 565(7737):101–5. doi: 10.1038/s41586-018-0806-7 PMC6420879 30568299

[7] Farmer JR Allard-Chamard H Sun N Ahmad M Bertocchi A Mahajan VS . Induction of metabolic quiescence defines the transitional to follicular b cell switch. Sci Signal (2019) 12(604):eaaw5573. doi: 10.1126/scisignal.aaw5573 31641080 PMC7301641

[8] Ersching J Efeyan A Mesin L Jacobsen JT Pasqual G Grabiner BC . Germinal center selection and affinity maturation require dynamic regulation of mTORC1 kinase. Immunity (2017) 46(6):1045–58.e6. doi: 10.1016/j.immuni.2017.06.005 28636954 PMC5526448

[9] Li M Lazorchak AS Ouyang X Zhang H Liu H Arojo OA . Sin1/mTORC2 regulate b cell growth and metabolism by activating mTORC1 and myc. Cell Mol Immunol (2019) 16(9):757–69. doi: 10.1038/s41423-018-0185-x PMC6804816 30705387

[10] Gualdoni GA Mayer KA Goschl L Boucheron N Ellmeier W Zlabinger GJ . The AMP analog AICAR modulates the Treg/Th17 axis through enhancement of fatty acid oxidation. FASEB J (2016) 30(11):3800–9. doi: 10.1096/fj.201600522R 27492924

[11] Wen Z Jin K Shen Y Yang Z Li Y Wu B . N-myristoyltransferase deficiency impairs activation of kinase AMPK and promotes synovial tissue inflammation. Nat Immunol (2019) 20(3):313–25. doi: 10.1038/s41590-018-0296-7 PMC6396296 30718913

[12] Sukumar M Liu J Mehta GU Patel SJ Roychoudhuri R Crompton JG . Mitochondrial membrane potential identifies cells with enhanced stemness for cellular therapy. Cell Metab (2016) 23(1):63–76. doi: 10.1016/j.cmet.2015.11.002 26674251 PMC4747432

[13] Wang Y Tao A Vaeth M Feske S . Calcium regulation of T cell metabolism. Curr Opin Physiol (2020) 17:207–23. doi: 10.1016/j.cophys.2020.07.016 PMC7584116 33103016

[14] Stolarczyk E Vong CT Perucha E Jackson I Cawthorne MA Wargent ET . Improved insulin sensitivity despite increased visceral adiposity in mice deficient for the immune cell transcription factor T-bet. Cell Metab (2013) 17(4):520–33. doi: 10.1016/j.cmet.2013.02.019 PMC3685808 23562076

[15] Johnson MO Wolf MM Madden MZ Andrejeva G Sugiura A Contreras DC . Distinct regulation of Th17 and Th1 cell differentiation by glutaminase-dependent metabolism. Cell (2018) 175(7):1780–95.e19. doi: 10.1016/j.cell.2018.10.001 30392958 PMC6361668

[16] Castellanos CA Ren X Gonzalez SL Li HK Schroeder AW Liang HE . Lymph node-resident dendritic cells drive TH2 cell development involving MARCH1. Sci Immunol (2021) 6(64):eabh0707. doi: 10.1126/sciimmunol.abh0707 34652961 PMC8736284

[17] Xia X Cao G Sun G Zhu L Tian Y Song Y . GLS1-mediated glutaminolysis unbridled by MALT1 protease promotes psoriasis pathogenesis. J Clin Invest (2020) 130(10):5180–96. doi: 10.1172/JCI129269 PMC7524468 32831293

[18] Danileviciute E Zeng N Capelle CM Paczia N Gillespie MA Kurniawan H . PARK7/DJ-1 promotes pyruvate dehydrogenase activity and maintains treg homeostasis during ageing. Nat Metab (2022) 4(5):589–607. doi: 10.1038/s42255-022-00576-y 35618940

[19] Brucklacher-Waldert V Ferreira C Stebegg M Fesneau O Innocentin S Marie JC . Cellular stress in the context of an inflammatory environment supports TGF-beta-Independent T helper-17 differentiation. Cell Rep (2017) 19(11):2357–70. doi: 10.1016/j.celrep.2017.05.052 PMC5483510 28614720

[20] Dang EV Barbi J Yang HY Jinasena D Yu H Zheng Y . Control of T(H)17/T(reg) balance by hypoxia-inducible factor 1. Cell (2011) 146(5):772–84. doi: 10.1016/j.cell.2011.07.033 PMC3387678 21871655

[21] Kawabe T Sun SL Fujita T Yamaki S Asao A Takahashi T . Homeostatic proliferation of naive CD4+ T cells in mesenteric lymph nodes generates gut-tropic Th17 cells. J Immunol (2013) 190(11):5788–98. doi: 10.4049/jimmunol.1203111 23610141

[22] Wu L Hollinshead KER Hao Y Au C Kroehling L Ng C . Niche-selective inhibition of pathogenic Th17 cells by targeting metabolic redundancy. Cell (2020) 182(3):641–54.e20. doi: 10.1016/j.cell.2020.06.014 32615085 PMC7556360

[23] Chalmin F Mignot G Bruchard M Chevriaux A Vegran F Hichami A . Stat3 and gfi-1 transcription factors control Th17 cell immunosuppressive activity *via via* the regulation of ectonucleotidase expression. Immunity (2012) 36(3):362–73. doi: 10.1016/j.immuni.2011.12.019 22406269

[24] Hesse J Siekierka-Harreis M Steckel B Alter C Schallehn M Honke N . Profound inhibition of CD73-dependent formation of anti-inflammatory adenosine in b cells of SLE patients. EBioMedicine (2021) 73:103616. doi: 10.1016/j.ebiom.2021.103616 34666225 PMC8524755

[25] Nemazee D . Mechanisms of central tolerance for b cells. Nat Rev Immunol (2017) 17(5):281–94. doi: 10.1038/nri.2017.19 PMC5623591 28368006

[26] Taher TE Ong VH Bystrom J Hillion S Simon Q Denton CP . Association of defective regulation of autoreactive interleukin-6-Producing transitional b lymphocytes with disease in patients with systemic sclerosis. Arthritis Rheumatol (2018) 70(3):450–61. doi: 10.1002/art.40390 29193892

[27] Turner M Mee PJ Costello PS Williams O Price AA Duddy LP . Perinatal lethality and blocked b-cell development in mice lacking the tyrosine kinase syk. Nature (1995) 378(6554):298–302. doi: 10.1038/378298a0 7477352

[28] Mesin L Ersching J Victora GD . Germinal center b cell dynamics. Immunity (2016) 45(3):471–82. doi: 10.1016/j.immuni.2016.09.001 PMC5123673 27653600

[29] Shi LZ Wang R Huang G Vogel P Neale G Green DR . HIF1alpha-dependent glycolytic pathway orchestrates a metabolic checkpoint for the differentiation of TH17 and treg cells. J Exp Med (2011) 208(7):1367–76. doi: 10.1084/jem.20110278 PMC3135370 21708926

[30] Ron-Harel N Santos D Ghergurovich JM Sage PT Reddy A Lovitch SB . Mitochondrial biogenesis and proteome remodeling promote one-carbon metabolism for T cell activation. Cell Metab (2016) 24(1):104–17. doi: 10.1016/j.cmet.2016.06.007 PMC5330619 27411012

[31] Wu B Qiu J Zhao TV Wang Y Maeda T Goronzy IN . Succinyl-CoA ligase deficiency in pro-inflammatory and tissue-invasive T cells. Cell Metab (2020) 32(6):967–80 e5. doi: 10.1016/j.cmet.2020.10.025 33264602 PMC7755381

[32] Caza TN Fernandez DR Talaber G Oaks Z Haas M Madaio MP . HRES-1/Rab4-mediated depletion of Drp1 impairs mitochondrial homeostasis and represents a target for treatment in SLE. Ann Rheum Dis (2014) 73(10):1888–97. doi: 10.1136/annrheumdis-2013-203794 PMC4047212 23897774

[33] Apostolidis SA Rauen T Hedrich CM Tsokos GC Crispin JC . Protein phosphatase 2A enables expression of interleukin 17 (IL-17) through chromatin remodeling. J Biol Chem (2013) 288(37):26775–84. doi: 10.1074/jbc.M113.483743 PMC3772223 23918926

[34] Hsu HC Yang P Wang J Wu Q Myers R Chen J . Interleukin 17-producing T helper cells and interleukin 17 orchestrate autoreactive germinal center development in autoimmune BXD2 mice. Nat Immunol (2008) 9(2):166–75. doi: 10.1038/ni1552 18157131

[35] Pfeifle R Rothe T Ipseiz N Scherer HU Culemann S Harre U . Regulation of autoantibody activity by the IL-23-TH17 axis determines the onset of autoimmune disease. Nat Immunol (2017) 18(1):104–13. doi: 10.1038/ni.3579 PMC5164937 27820809

[36] Zeng QH Wei Y Lao XM Chen DP Huang CX Lin QY . B cells polarize pathogenic inflammatory T helper subsets through ICOSL-dependent glycolysis. Sci Adv (2020) 6(37):eabb6296. doi: 10.1126/sciadv.abb6296 32917682 PMC11206526

[37] Sun V Sharpley M Kaczor-Urbanowicz KE Chang P Montel-Hagen A Lopez S . The metabolic landscape of thymic T cell development *In VivoIn vivo* and in VitroIn vitro. Front Immunol (2021) 12:716661. doi: 10.3389/fimmu.2021.716661 34394122 PMC8355594

[38] Palmer DB . The effect of age on thymic function. Front Immunol (2013) 4:316. doi: 10.3389/fimmu.2013.00316 24109481 PMC3791471

[39] Baricza E Marton N Kiralyhidi P Kovacs OT Kovacsne Szekely I Lajko E . Distinct *In VitroIn vitro* T-helper 17 differentiation capacity of peripheral naive T cells in rheumatoid and psoriatic arthritis. Front Immunol (2018) 9:606. doi: 10.3389/fimmu.2018.00606 29670615 PMC5893718

[40] Mukasa R Balasubramani A Lee YK Whitley SK Weaver BT Shibata Y . Epigenetic instability of cytokine and transcription factor gene loci underlies plasticity of the T helper 17 cell lineage. Immunity (2010) 32(5):616–27. doi: 10.1016/j.immuni.2010.04.016 PMC3129685 20471290

[41] Heikamp EB Patel CH Collins S Waickman A Oh MH Sun IH . The AGC kinase SGK1 regulates TH1 and TH2 differentiation downstream of the mTORC2 complex. Nat Immunol (2014) 15(5):457–64. doi: 10.1038/ni.2867 PMC4267697 24705297

[42] Sun IH Oh MH Zhao L Patel CH Arwood ML Xu W . mTOR complex 1 signaling regulates the generation and function of central and effector Foxp3(+) regulatory T cells. J Immunol (2018) 201(2):481–92. doi: 10.4049/jimmunol.1701477 PMC6089237 29884702

[43] De Rosa V Galgani M Porcellini A Colamatteo A Santopaolo M Zuchegna C . Glycolysis controls the induction of human regulatory T cells by modulating the expression of FOXP3 exon 2 splicing variants. Nat Immunol (2015) 16(11):1174–84. doi: 10.1038/ni.3269 PMC4868085 26414764

[44] Gerriets VA Kishton RJ Johnson MO Cohen S Siska PJ Nichols AG . Foxp3 and toll-like receptor signaling balance treg cell anabolic metabolism for suppression. Nat Immunol (2016) 17(12):1459–66. doi: 10.1038/ni.3577 PMC5215903 27695003

[45] Mayberry CL Logan NA Wilson JJ Chang CH . Providing a helping hand: Metabolic regulation of T follicular helper cells and their association with disease. Front Immunol (2022) 13:864949. doi: 10.3389/fimmu.2022.864949 35493515 PMC9047778

[46] Omenetti S Bussi C Metidji A Iseppon A Lee S Tolaini M . The intestine harbors functionally distinct homeostatic tissue-resident and inflammatory Th17 cells. Immunity (2019) 51(1):77–89.e6. doi: 10.1016/j.immuni.2019.05.004 31229354 PMC6642154

[47] Kahlfuss S Kaufmann U Concepcion AR Noyer L Raphael D Vaeth M . STIM1-mediated calcium influx controls antifungal immunity and the metabolic function of non-pathogenic Th17 cells. EMBO Mol Med (2020) 12(8):e11592. doi: 10.15252/emmm.201911592 32609955 PMC7411566

[48] Shin B Benavides GA Geng J Koralov SB Hu H Darley-Usmar VM . Mitochondrial oxidative phosphorylation regulates the fate decision between pathogenic Th17 and regulatory T cells. Cell Rep (2020) 30(6):1898–909 e4. doi: 10.1016/j.celrep.2020.01.022 32049019 PMC9059282

[49] Delgoffe GM Pollizzi KN Waickman AT Heikamp E Meyers DJ Horton MR . The kinase mTOR regulates the differentiation of helper T cells through the selective activation of signaling by mTORC1 and mTORC2. Nat Immunol (2011) 12(4):295–303. doi: 10.1038/ni.2005 21358638 PMC3077821

[50] Gerriets VA Kishton RJ Nichols AG Macintyre AN Inoue M Ilkayeva O . Metabolic programming and PDHK1 control CD4+ T cell subsets and inflammation. J Clin Invest (2015) 125(1):194–207. doi: 10.1172/JCI76012 25437876 PMC4382238

[51] Xu T Stewart KM Wang X Liu K Xie M Ryu JK . Metabolic control of TH17 and induced treg cell balance by an epigenetic mechanism. Nature (2017) 548(7666):228–33. doi: 10.1038/nature23475 PMC6701955 28783731

[52] Mak TW Grusdat M Duncan GS Dostert C Nonnenmacher Y Cox M . Glutathione primes T cell metabolism for inflammation. Immunity (2017) 46(4):675–89. doi: 10.1016/j.immuni.2017.03.019 28423341

[53] Roy DG Chen J Mamane V Ma EH Muhire BM Sheldon RD . Methionine metabolism shapes T helper cell responses through regulation of epigenetic reprogramming. Cell Metab (2020) 31(2):250–66 e9. doi: 10.1016/j.cmet.2020.01.006 32023446

[54] Berod L Friedrich C Nandan A Freitag J Hagemann S Harmrolfs K . *De novoDe novo* fatty acid synthesis controls the fate between regulatory T and T helper 17 cells. Nat Med (2014) 20(11):1327–33. doi: 10.1038/nm.3704 25282359

[55] Sundrud MS Koralov SB Feuerer M Calado DP Kozhaya AE Rhule-Smith A . Halofuginone inhibits TH17 cell differentiation by activating the amino acid starvation response. Science (2009) 324(5932):1334–8. doi: 10.1126/science.1172638 PMC2803727 19498172

[56] Kleinewietfeld M Manzel A Titze J Kvakan H Yosef N Linker RA . Sodium chloride drives autoimmune disease by the induction of pathogenic TH17 cells. Nature (2013) 496(7446):518–22. doi: 10.1038/nature11868 PMC3746493 23467095

[57] Zhang D Jin W Wu R Li J Park SA Tu E . High glucose intake exacerbates autoimmunity through reactive-Oxygen-Species-Mediated TGF-beta cytokine activation. Immunity (2019) 51(4):671–81.e5. doi: 10.1016/j.immuni.2019.08.001 31451397 PMC9811990

[58] Laurence A Tato CM Davidson TS Kanno Y Chen Z Yao Z . Interleukin-2 signaling *via via* STAT5 constrains T helper 17 cell generation. Immunity (2007) 26(3):371–81. doi: 10.1016/j.immuni.2007.02.009 17363300

[59] Revu S Wu J Henkel M Rittenhouse N Menk A Delgoffe GM . IL-23 and IL-1beta drive human Th17 cell differentiation and metabolic reprogramming in absence of CD28 costimulation. Cell Rep (2018) 22(10):2642–53. doi: 10.1016/j.celrep.2018.02.044 PMC5884137 29514093

[60] Sugiura A Andrejeva G Voss K Heintzman DR Xu X Madden MZ . MTHFD2 is a metabolic checkpoint controlling effector and regulatory T cell fate and function. Immunity (2022) 55(1):65–81.e9. doi: 10.1016/j.immuni.2021.10.011 34767747 PMC8755618

[61] Patsoukis N Bardhan K Chatterjee P Sari D Liu B Bell LN . PD-1 alters T-cell metabolic reprogramming by inhibiting glycolysis and promoting lipolysis and fatty acid oxidation. Nat Commun (2015) 6:6692. doi: 10.1038/ncomms7692 25809635 PMC4389235

[62] Wagner A Wang C Fessler J DeTomaso D Avila-Pacheco J Kaminski J . Metabolic modeling of single Th17 cells reveals regulators of autoimmunity. Cell (2021) 184(16):4168–85.e21. doi: 10.1016/j.cell.2021.05.045 34216539 PMC8621950

[63] Carriche GM Almeida L Stuve P Velasquez L Dhillon-LaBrooy A Roy U . Regulating T-cell differentiation through the polyamine spermidine. J Allergy Clin Immunol (2021) 147(1):335–48 e11. doi: 10.1016/j.jaci.2020.04.037 32407834

[64] Clarke AJ Riffelmacher T Braas D Cornall RJ Simon AK . B1a b cells require autophagy for metabolic homeostasis and self-renewal. J Exp Med (2018) 215(2):399–413. doi: 10.1084/jem.20170771 29326381 PMC5789411

[65] Iperi C Bordron A Dueymes M Pers JO Jamin C . Metabolic program of regulatory b lymphocytes and influence in the control of malignant and autoimmune situations. Front Immunol (2021) 12:735463. doi: 10.3389/fimmu.2021.735463 34650560 PMC8505885

[66] Kojima H Kobayashi A Sakurai D Kanno Y Hase H Takahashi R . Differentiation stage-specific requirement in hypoxia-inducible factor-1alpha-regulated glycolytic pathway during murine b cell development in bone marrow. J Immunol (2010) 184(1):154–63. doi: 10.4049/jimmunol.0800167 PMC2848717 19949104

[67] Stein M Dutting S Mougiakakos D Bosl M Fritsch K Reimer D . A defined metabolic state in pre b cells governs b-cell development and is counterbalanced by swiprosin-2/EFhd1. Cell Death Differ (2017) 24(7):1239–52. doi: 10.1038/cdd.2017.52 PMC5520169 28524857

[68] Akkaya M Pierce SK . From zero to sixty and back to zero again: the metabolic life of b cells. Curr Opin Immunol (2019) 57:1–7. doi: 10.1016/j.coi.2018.09.019 30312894 PMC6456432

[69] Herzog S Hug E Meixlsperger S Paik JH DePinho RA Reth M . SLP-65 regulates immunoglobulin light chain gene recombination through the PI(3)K-PKB-Foxo pathway. Nat Immunol (2008) 9(6):623–31. doi: 10.1038/ni.1616 18488031

[70] Adams WC Chen YH Kratchmarov R Yen B Nish SA Lin WW . Anabolism-associated mitochondrial stasis driving lymphocyte differentiation over self-renewal. Cell Rep (2016) 17(12):3142–52. doi: 10.1016/j.celrep.2016.11.065 PMC5189677 28009285

[71] Jellusova J Cato MH Apgar JR Ramezani-Rad P Leung CR Chen C . Gsk3 is a metabolic checkpoint regulator in b cells. Nat Immunol (2017) 18(3):303–12. doi: 10.1038/ni.3664 PMC5310963 28114292

[72] Chan LN Chen Z Braas D Lee JW Xiao G Geng H . Metabolic gatekeeper function of b-lymphoid transcription factors. Nature (2017) 542(7642):479–83. doi: 10.1038/nature21076 PMC5621518 28192788

[73] Waters LR Ahsan FM Wolf DM Shirihai O Teitell MA . Initial b cell activation induces metabolic reprogramming and mitochondrial remodeling. iScience (2018) 5:99–109. doi: 10.1016/j.isci.2018.07.005 30240649 PMC6123864

[74] Akkaya M Traba J Roesler AS Miozzo P Akkaya B Theall BP . Second signals rescue b cells from activation-induced mitochondrial dysfunction and death. Nat Immunol (2018) 19(8):871–84. doi: 10.1038/s41590-018-0156-5 PMC6202187 29988090

[75] Ise W Fujii K Shiroguchi K Ito A Kometani K Takeda K . T Follicular helper cell-germinal center b cell interaction strength regulates entry into plasma cell or recycling germinal center cell fate. Immunity (2018) 48(4):702–15 e4. doi: 10.1016/j.immuni.2018.03.027 29669250

[76] Weisel FJ Mullett SJ Elsner RA Menk AV Trivedi N Luo W . Germinal center b cells selectively oxidize fatty acids for energy while conducting minimal glycolysis. Nat Immunol (2020) 21(3):331–42. doi: 10.1038/s41590-020-0598-4 PMC7112716 32066950

[77] Tsui C Martinez-Martin N Gaya M Maldonado P Llorian M Legrave NM . Protein kinase c-beta dictates b cell fate by regulating mitochondrial remodeling, metabolic reprogramming, and heme biosynthesis. Immunity (2018) 48(6):1144–59 e5. doi: 10.1016/j.immuni.2018.04.031 29884460 PMC6015119

[78] Lam WY Becker AM Kennerly KM Wong R Curtis JD Llufrio EM . Mitochondrial pyruvate import promotes long-term survival of antibody-secreting plasma cells. Immunity (2016) 45(1):60–73. doi: 10.1016/j.immuni.2016.06.011 27396958 PMC4956536

[79] Rubtsov AV Swanson CL Troy S Strauch P Pelanda R Torres RM . TLR agonists promote marginal zone b cell activation and facilitate T-dependent IgM responses. J Immunol (2008) 180(6):3882–8. doi: 10.4049/jimmunol.180.6.3882 18322196

[80] Sintes J Gentile M Zhang S Garcia-Carmona Y Magri G Cassis L . mTOR intersects antibody-inducing signals from TACI in marginal zone b cells. Nat Commun (2017) 8(1):1462. doi: 10.1038/s41467-017-01602-4 29133782 PMC5684130

[81] Lal G Kulkarni N Nakayama Y Singh AK Sethi A Burrell BE . IL-10 from marginal zone precursor b cells controls the differentiation of Th17, tfh and tfr cells in transplantation tolerance. Immunol Lett (2016) 170:52–63. doi: 10.1016/j.imlet.2016.01.002 26772435 PMC4740190

[82] Sano T Kageyama T Fang V Kedmi R Martinez CS Talbot J . Redundant cytokine requirement for intestinal microbiota-induced Th17 cell differentiation in draining lymph nodes. Cell Rep (2021) 36(8):109608. doi: 10.1016/j.celrep.2021.109608 34433045 PMC8845566

[83] Luu M Pautz S Kohl V Singh R Romero R Lucas S . The short-chain fatty acid pentanoate suppresses autoimmunity by modulating the metabolic-epigenetic crosstalk in lymphocytes. Nat Commun (2019) 10(1):760. doi: 10.1038/s41467-019-08711-2 30770822 PMC6377655

[84] Amezcua Vesely MC Pallis P Bielecki P Low JS Zhao J Harman CCD . Effector TH17 cells give rise to long-lived TRM cells that are essential for an immediate response against bacterial infection. Cell (2019) 178(5):1176–88 e15. doi: 10.1016/j.cell.2019.07.032 31442406 PMC7057720

[85] Kirchner FR LeibundGut-Landmann S . Tissue-resident memory Th17 cells maintain stable fungal commensalism in the oral mucosa. Mucosal Immunol (2021) 14(2):455–67. doi: 10.1038/s41385-020-0327-1 PMC7946631 32719409

[86] Langley RG Elewski BE Lebwohl M Reich K Griffiths CE Papp K . Secukinumab in plaque psoriasis–results of two phase 3 trials. N Engl J Med (2014) 371(4):326–38. doi: 10.1056/NEJMoa1314258 25007392

[87] Kokkonen H Soderstrom I Rocklov J Hallmans G Lejon K Rantapaa Dahlqvist S . Up-regulation of cytokines and chemokines predates the onset of rheumatoid arthritis. Arthritis Rheumatol (2010) 62(2):383–91. doi: 10.1002/art.27186 20112361

[88] Yang Z Fujii H Mohan SV Goronzy JJ Weyand CM . Phosphofructokinase deficiency impairs ATP generation, autophagy, and redox balance in rheumatoid arthritis T cells. J Exp Med (2013) 210(10):2119–34. doi: 10.1084/jem.20130252 PMC3782046 24043759

[89] Qiu J Wu B Goodman SB Berry GJ Goronzy JJ Weyand CM . Metabolic control of autoimmunity and tissue inflammation in rheumatoid arthritis. Front Immunol (2021) 12:652771. doi: 10.3389/fimmu.2021.652771 33868292 PMC8050350

[90] Ueda Y Saegusa J Okano T Sendo S Yamada H Nishimura K . Additive effects of inhibiting both mTOR and glutamine metabolism on the arthritis in SKG mice. Sci Rep (2019) 9(1):6374. doi: 10.1038/s41598-019-42932-1 31011190 PMC6476881

[91] Cervantes-Madrid D Romero Y Duenas-Gonzalez A . Reviving lonidamine and 6-Diazo-5-oxo-L-norleucine to be used in combination for metabolic cancer therapy. BioMed Res Int (2015) 2015:690492. doi: 10.1155/2015/690492 26425550 PMC4575731

[92] Pucino V Certo M Bulusu V Cucchi D Goldmann K Pontarini E . Lactate buildup at the site of chronic inflammation promotes disease by inducing CD4(+) T cell metabolic rewiring. Cell Metab (2019) 30(6):1055–74 e8. doi: 10.1016/j.cmet.2019.10.004 31708446 PMC6899510

[93] Miossec P . Local and systemic effects of IL-17 in joint inflammation: a historical perspective from discovery to targeting. Cell Mol Immunol (2021) 18(4):860–5. doi: 10.1038/s41423-021-00644-5 PMC7943939 33692481

[94] Wu B Zhao TV Jin K Hu Z Abdel MP Warrington KJ . Mitochondrial aspartate regulates TNF biogenesis and autoimmune tissue inflammation. Nat Immunol (2021) 22(12):1551–62. doi: 10.1038/s41590-021-01065-2 PMC8756813 34811544

[95] Bystrom J Clanchy FI Taher TE Al-Bogami MM Muhammad HA Alzabin S . Response to treatment with TNFalpha inhibitors in rheumatoid arthritis is associated with high levels of GM-CSF and GM-CSF(+) T lymphocytes. Clin Rev Allergy Immunol (2017) 53(2):265–76. doi: 10.1007/s12016-017-8610-y PMC5597702 28488248

[96] Alzabin S Abraham SM Taher TE Palfreeman A Hull D McNamee K . Incomplete response of inflammatory arthritis to TNFalpha blockade is associated with the Th17 pathway. Ann Rheum Dis (2012) 71(10):1741–8. doi: 10.1136/annrheumdis-2011-201024 22550316

[97] Yin Y Choi SC Xu Z Perry DJ Seay H Croker BP . Normalization of CD4+ T cell metabolism reverses lupus. Sci Transl Med (2015) 7(274):274ra18. doi: 10.1126/scitranslmed.aaa0835 PMC5292723 25673763

[98] Godavarthy A Kelly R Jimah J Beckford M Caza T Fernandez D . Lupus-associated endogenous retroviral LTR polymorphism and epigenetic imprinting promote HRES-1/RAB4 expression and mTOR activation. JCI Insight (2020) 5(1):e134010. doi: 10.1172/jci.insight.134010 PMC7030820 31805010

[99] Vogelzang A McGuire HM Yu D Sprent J Mackay CR King C . A fundamental role for interleukin-21 in the generation of T follicular helper cells. Immunity (2008) 29(1):127–37. doi: 10.1016/j.immuni.2008.06.001 18602282

[100] Frasca D Romero M Garcia D Diaz A Blomberg BB . Hyper-metabolic b cells in the spleens of old mice make antibodies with autoimmune specificities. Immun Ageing (2021) 18(1):9. doi: 10.1186/s12979-021-00222-3 33639971 PMC7916295

[101] Jing Y Luo L Chen Y Westerberg LS Zhou P Xu Z . SARS-CoV-2 infection causes immunodeficiency in recovered patients by downregulating CD19 expression in b cells *via via* enhancing b-cell metabolism. Signal Transduct Target Ther (2021) 6(1):345. doi: 10.1038/s41392-021-00749-3 34552055 PMC8456405

[102] Frasca D Pallikkuth S Pahwa S . Metabolic phenotype of b cells from young and elderly HIV individuals. Immun Ageing (2021) 18(1):35. doi: 10.1186/s12979-021-00245-w 34419088 PMC8380009

[103] Tipton CM Hom JR Fucile CF Rosenberg AF Sanz I . Understanding b-cell activation and autoantibody repertoire selection in systemic lupus erythematosus: A b-cell immunomics approach. Immunol Rev (2018) 284(1):120–31. doi: 10.1111/imr.12660 PMC6022284 29944759

[104] Lee DM Schur PH . Clinical utility of the anti-CCP assay in patients with rheumatic diseases. Ann Rheum Dis (2003) 62(9):870–4. doi: 10.1136/ard.62.9.870 PMC1754666 12922961

[105] Floudas A Neto N Marzaioli V Murray K Moran B Monaghan MG . Pathogenic, glycolytic PD-1+ b cells accumulate in the hypoxic RA joint. JCI Insight (2020) 5(21):e139032. doi: 10.1172/jci.insight.139032 PMC7710281 33148884

[106] Torigoe M Iwata S Nakayamada S Sakata K Zhang M Hajime M . Metabolic reprogramming commits differentiation of human CD27(+)IgD(+) b cells to plasmablasts or CD27(-)IgD(-) cells. J Immunol (2017) 199(2):425–34. doi: 10.4049/jimmunol.1601908 28626065

[107] Taylor JJ Pape KA Jenkins MK . A germinal center-independent pathway generates unswitched memory b cells early in the primary response. J Exp Med (2012) 209(3):597–606. doi: 10.1084/jem.20111696 22370719 PMC3302224

[108] Lee SY Moon SJ Kim EK Seo HB Yang EJ Son HJ . Metformin suppresses systemic autoimmunity in roquin(san/san) mice through inhibiting b cell differentiation into plasma cells *via via* regulation of AMPK/mTOR/STAT3. J Immunol (2017) 198(7):2661–70. doi: 10.4049/jimmunol.1403088 PMC5357783 28242651

[109] Paulos CM Carpenito C Plesa G Suhoski MM Varela-Rohena A Golovina TN . The inducible costimulator (ICOS) is critical for the development of human T(H)17 cells. Sci Transl Med (2010) 2(55):55ra78. doi: 10.1126/scitranslmed.3000448 PMC6282816 20980695

